# PNAUM: integrated approach to Pharmaceutical Services, Science, Technology and Innovation

**DOI:** 10.1590/S1518-8787.2016050006153

**Published:** 2016-12-01

**Authors:** Carlos Augusto Grabois Gadelha, Karen Sarmento Costa, José Miguel do Nascimento, Orlando Mário Soeiro, Sotero Serrate Mengue, Márcia Luz da Motta, Antônio Carlos Campos de Carvalho

**Affiliations:** I Departamento de Administração e Planejamento em Saúde. Escola Nacional de Saúde Pública Sérgio Arouca. Fundação Oswaldo Cruz. Rio de Janeiro, RJ, Brasil; II Núcleo de Estudos de Políticas Públicas. Universidade de Campinas. Campinas, SP, Brasil; III Secretaria Municipal de Saúde. Prefeitura Municipal de Florianópolis. Florianópolis, SC, Brasil; IV Departamento de Assistência Farmacêutica e Insumos Estratégicos. Secretaria de Ciência, Tecnologia e Insumos Estratégicos. Ministério da Saúde. Brasília, DF, Brasil; V Programa de Pós-Graduação em Epidemiologia. Faculdade de Medicina. Universidade Federal do Rio Grande do Sul. Porto Alegre, RS, Brasil; VI Gabinete da Fiocruz Brasília. Fundação Oswaldo Cruz. Brasília, DF, Brasil; VII Instituto de Biofísica Carlos Chagas Filho. Universidade Federal do Rio de Janeiro. Rio de Janeiro, RJ, Brasil

**Keywords:** National Policy of Pharmaceutical Services, Innovation and Development Policy, Public Health Policy, Unified Health System

## Abstract

This paper describes the development process of the *Pesquisa Nacional sobre Acesso, Utilização e Promoção do Uso Racional de Medicamentos* (PNAUM – National Survey on Access, Use and Promotion of Rational Use of Medicines) based on an integrated approach to pharmaceutical services, science, technology and innovation. It starts by contextualizing health and development in Brazil and features elements of the National Policy for Science, Technology and Innovation in Health in Brazil and the National Policy for Pharmaceutical Services. On presenting pharmaceutical policy guidelines, it stresses the lack of nationwide data. This survey, commissioned by the Brazilian Ministry of Health, has two components: household survey and evaluation of pharmaceutical services in primary care. The findings point to perspectives that represent, besides the enhancement of public policy for pharmaceutical services and public health, results of government action aimed at developing the economic and industrial health care complex to improve the health conditions of the Brazilian population.

## RESUMO

O artigo apresenta o processo de construção da Pesquisa Nacional sobre Acesso, Utilização e Promoção do Uso Racional de Medicamento a partir de uma concepção integradora da Assistência Farmacêutica, Ciência, Tecnologia e Inovação. Inicia-se contextualizando a saúde e o desenvolvimento no País e apresenta elementos da Política Nacional de Ciência Tecnologia e Inovação em Saúde no Brasil e da Política Nacional de Assistência Farmacêutica. Ao apresentar as diretrizes das Políticas Farmacêuticas, destaca-se a carência de dados de abrangência nacional. A presente pesquisa, encomendada pelo Ministério da Saúde, foi estruturada em dois componentes: inquérito domiciliar e avaliação dos serviços de assistência farmacêutica na atenção básica. As perspectivas dos resultados representam, além do incremento das políticas públicas farmacêuticas e de saúde pública, resultados de ações governamentais voltadas ao desenvolvimento do complexo econômico-industrial da saúde, visando a melhoria das condições de saúde da população brasileira.

## INTRODUCTION

This supplement of *Revista de Saúde Pública* (Journal of Public Health) is a milestone in the field of pharmaceutical public policy in Brazil. It presents the results of the first nationwide survey on the subject and addresses the interface between pharmaceutical policy and policy for science, technology and innovation in health in Brazil. The Secretariat of Science, Technology and Strategic Inputs of the Brazilian Ministry of Health commissioned from research institutions of different Brazilian state and federal public universities the *Pesquisa Nacional sobre Acesso, Utilização e Promoção do Uso Racional de Medicamentos* (PNAUM – National Survey on Access, Use and Promotion of Rational Use of Medicines).

The opening article of this unprecedented and rich compilation features the development process of PNAUM based on an integrated approach to pharmaceutical services, science, technology and innovation. It begins by discussing the background of health development and of the National Policy for Science, Technology and Innovation in Health in Brazil; it presents a brief overview of research, science and innovation in health and the role of the Departments of Science and Technology (DECIT) and Pharmaceutical Services and Strategic Health Supplies (DAF), as well as the design process of this research and the perspectives of the Brazilian Ministry of Health for the development of strategies for pharmaceutical policy.

## HEALTH AND DEVELOPMENT

The theme of health has been institutionalized and incorporated into the national development agenda, in the field of science, technology and innovation, because of its strategic nature. Health is an inherent component of the social dimension of development, for several reasons: it enables the care of human needs; it plays a significant role in the country’s economic development, generating employment and income with 14 million direct and five million indirect jobs, which, in 2009, accounted for approximately 9.0% of the gross domestic product; and it holds a leading position in terms of investment in research and development^2^. Thus, health is not only part of the welfare state and a basic condition of citizenship for the Brazilian population, but it also helps to generate employment, income and wealth in Brazil^3^.

Concerning research and development, much of the effort has been made in the field of health, integrated worldwide to a set of technologies related to an international and competitive standard of knowledge: nanotechnology, biotechnology, information and communication technology, among others^3^. In Brazil, public investment in research and development in health has reached significant levels since the organization of the 2nd National Conference on Science, Technology and Innovation in Health in 2004. The health sector is the largest component of scientific and technological production in Brazil and several countries^4^.

In the social and economic life of nations, the incorporation of the advances in science, technology and innovation into the health sector contributes decisively to improve the living conditions of the population^2^.

## LEGAL AND REGULATORY FRAMEWORK OF THE NATIONAL POLICY FOR SCIENCE, TECHNOLOGY AND INNOVATION IN HEALTH IN BRAZIL

Article 200, section V of the 1988 Federal Constitution establishes the competence of the Brazilian health system, which includes fostering the development of scientific and technological development in health. The National Policy for Science, Technology and Innovation in Health was formulated in 2004 as part of the National Health Policy of the Brazilian Unified Health System (SUS)^4^, and of the National Policy for Science, Technology and Innovation, being subject to the same principles that govern the latter: technical-scientific merit and social relevance^6^ ^,^ ^a^.

The approval of the National Policy for Science, Technology and Innovation in Health in 2004 was the result of a collective consensus on the relevance of implementing initiatives to foster science, technology and innovation, linked to SUS needs, in order to improve the fragile integration between support initiatives in science, technology and innovation and health policy. This collective effort involved, in all stages, fifteen thousand participants from the fields of health, science and technology, and education^4^.

The objective of the National Policy for Science, Technology and Innovation in Health, as well as of the National Policy for Science, Technology and Innovation, is to contribute to sustainable national development via the production of scientific and technological knowledge linked to the country’s economic, social, cultural and political needs^4^.

The creation of the Secretariat of Science, Technology and Strategic Inputs (SCTIE) in 2003 led to the gradual and progressive incorporation into public health policy of issues and demands hitherto discussed in the areas of science, technology and innovation; on the other hand, it established more active interaction with the field of health. Thus, the creation of a secretariat with such a profile was a decisive factor in implementing the National Policy for Science, Technology and Innovation in Health^4^.

**Figure 1:**
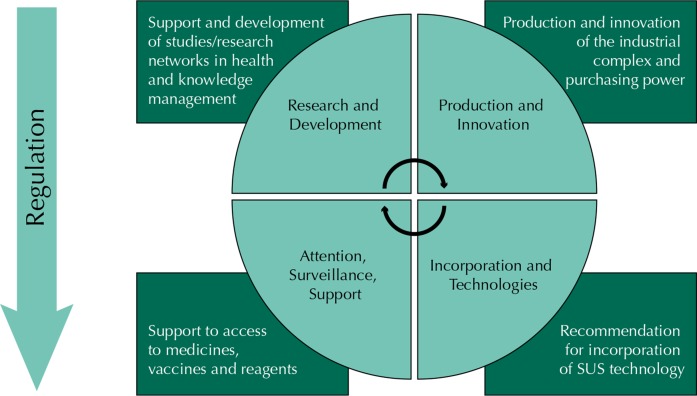
Science, Technology and Innovation within the Brazilian Ministry of Health.

The SCTIE is divided into four departments: Pharmaceutical Services and Strategic Health Supplies, Science and Technology, Industrial Complex and Innovation in Health, and, more recently, Management and Incorporation of Health Tecnology^a^. The departments jointly seek to develop programs and activities by applying science, technology and innovation to the field of health, which comprises an intrinsic relationship between research and development; production and innovation; incorporation of technologies; health care, surveillance and support, as shown in Figure 1.

## EVOLUTION OF SCIENTIFIC PRODUCTIVITY AND INCENTIVE TO SCIENCE, TECHNOLOGY AND INNOVATION IN HEALTH IN BRAZIL

According to SCImago Journal & Country Rank, in 2014 Brazil ranked 14th worldwide in absolute number of scientific articles on health published in journals indexed in the Scopus® database. As for scientific production in health between 1996 and 2014 among the BRICS countries (Brazil, Russia, India, China and South Africa), Brazil ranked 3rd, behind China and India^b^. In addition, scientific production in health in Brazil has grown significantly in the last two decades. In 1996, Brazil accounted for approximately 40.2% and 0.7% of scientific production in health in Latin America and worldwide, respectively. However, in 2014, these percentages increased to 55.2% and 2.2%, respectively^c^.

**Figure 2:**
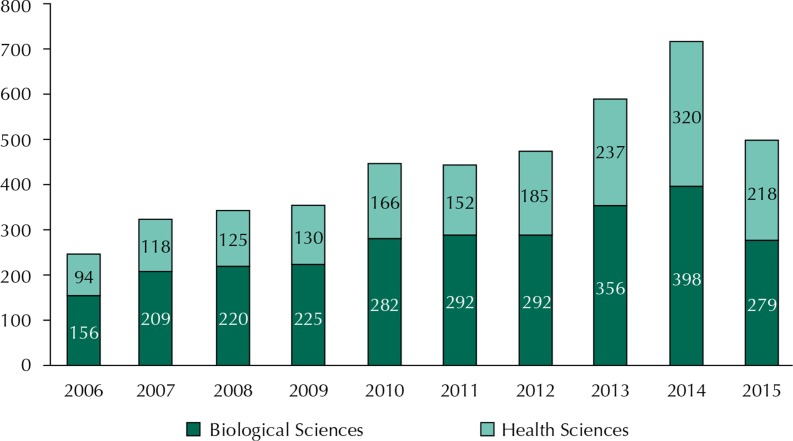
Evolution of CNPq/MCTI disbursements* to support research (grants in Brazil + grants abroad + research funding) in Health and Biological Sciences in millions of reais.

Source: Conselho Nacional de Desenvolvimento Científico e Tecnológico. CNPq investment panel. Brasília (DF); 2015 [cited 2015 Nov 24]. Available from: http://www.cnpq.br/painel-de-investimentos

Regarding support to health research in Brazil, disbursements in science, technology and innovation increased significantly in the last decade, totaling approximately R$4.5 billion (Figure 2).

* Disbursements in 2015 between January 1 and November 24.

## THE ROLE OF DECIT FUNDING IN HEALTH RESEARCH IN BRAZIL

Since the creation of the Department of Science and Technology (DECIT) – by Decree 3496 of June 1, 2000 – the Brazilian Ministry of Health has made significant advances in supporting health research in Brazil, as well as in formulating, implementing and evaluating the National Policy on Science, Technology and Innovation in Health, aiming to use scientific and technological knowledge at all SUS management levels. The research support modalities used by DECIT include national research public calls, decentralized funding (Research Program for the Unified Health System: Shared Management in Health), and direct financing. Between 2011 and 2015, 1,514 projects were funded, totaling approximately R$292 million invested by this department and its partners^d^.

The national and international partnerships established by DECIT – such as Brazilian Development Bank, Coordination for the Improvement of Higher Education Personnel, Funding Authority for Studies and Projects, National Council for Scientific and Technological Development, Brazilian State Funding Agencies, State Health Departments, Bill & Melinda Gates Foundation, National Institute of Health, *Agencia Argentina de Promoción Científica y Tecnológica*, among others – has allowed increased investment by other institutions and the development of scientific cooperation networks at national and international level in strategic and priority research for the Brazilian Ministry of Health, aimed at strengthening SUS.

## DECIT SUPPORT IN PHARMACEUTICAL SERVICES

All DECIT support initiatives aim to fund research to strengthen the principles of SUS – the promotion of universality, equity, integrality and resoluteness. As for pharmaceutical services, via national and state (Research Program for the Unified Health System: Shared Management in Health) public notices and direct funding, DECIT financed approximately 228 studies in 2011-2014, totaling R$33.5 million.

Acknowledging that the country lacked a national population-based survey to specifically identify aspects related to the National Policy for Pharmaceutical Services (PNAF)^e^, SCTIE invested more than R$9.4 million in PNAUM.

## THE ROLE OF THE DEPARTMENT FOR PHARMACEUTICAL SERVICES AND STRATEGIC HEALTH SUPPLIES IN NATIONAL PHARMACEUTICAL POLICY

In recent years, integrated care, guaranteed by the Organic Law on Health^f^, has been a focus of reflection and debate among managers, professionals, scholars in the field of health, the judiciary and other actors in society, regarding the principle of integrality, criteria to incorporate technology in health, and availability of medication for the population^5^.

To meet this requirement, pharmaceutical care was implemented as public policy at national level with the enactment of the National Medicines Policy^g^. This policy has the following main objectives: to ensure the safety, efficacy and quality of medicines; to promote the rational use of medicines; and to guarantee the population’s access to essential medicines^5^.

Improvements to the National Medicines Policy aimed to complement and enhance the proposals of pharmaceutical services in the country, in order to incorporate more explicitly the principles of universality, integrality and equity, and the initiatives for health promotion, protection and recovery established in SUS.

The PNAF was developed in 2004 based on an integrated approach, and is understood as public policy and an integral part of the National Health Policy^7^.

The resolution that enacted PNAF^e^ defines pharmaceutical services as a set of initiatives for health promotion, protection and recovery, individually and collectively, using medicines as an essential input, aiming at access and rational use. This set of initiatives involves “research, development and production of medicines and inputs, as well as their selection, planning, procurement, distribution, dispensing, guarantee of product and service quality, monitoring and evaluation of use, in order to achieve concrete results and improve the quality of life of the population” (p.52)^e^.

The creation of the Department for Pharmaceutical Services and Strategic Health Supplies in 2013 institutionalized a national sector to develop and coordinate the National Medicines Policy in Brazil. The department’s responsibilities include: cooperating technically to improve the managerial and operational capacity of states and municipalities; promoting, regulating and coordinating the procurement and distribution of strategic inputs, at different levels of care; supporting, monitoring and evaluating the implementation of pharmaceutical care services and enhancing the quality of pharmaceutical services in SUS.

The Brazilian government has developed various initiatives to implement these policies and guarantee the population’s access to pharmaceutical services and medicines. These include *Programa Farmácia Popular do Brasil* (Brazilian Popular Pharmacy Program), in 2004; *Política Nacional de Plantas Medicinais e Fitoterápicos* (National Policy for Medicinal Plants and Herbal Medicines), in 2006; *Componente Especializado da Assistência Farmacêutica* (Specialized Component for Pharmaceutical Services), in 2009; *Programa Nacional de Qualificação da Assistência Farmacêutica* (National Program of Pharmaceutical Services Qualification), within SUS (QUALIFAR-SUS), in 2012^h^, among others. Such policies are incentives for scientific, technological and innovation development in the Brazil.

**Figure 3:**
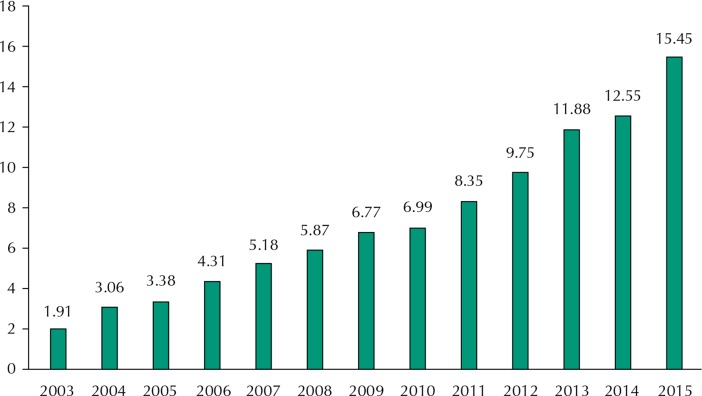
Evolution of disbursements (in billions of reais) by the Department of Pharmaceutical services and Strategic Inputs of the Brazilian Ministry of Health. Brazil, 2003-2005.

Source: Brazilian Ministry of Health. National Health Fund – FNS and CGPLAN/SCTIE/MS [cited 2015 Nov 20]. Available from: http://www.fns.saude.gov.br

In addition to public policies, programs and initiatives, the Brazilian Ministry of Health has provided and expanded investment in pharmaceutical services in the country, which increased from approximately R$2 billion in 2003 to around R$15 billion in 2015 (Figure 3).

Increasing investment in pharmaceutical policy in Brazil requires from public managers information and rational planning focused on implementing such policy. Therefore, it has become imperative to obtain data which is more accurate and representative of access to medicines and of their rational use by the population. In this context, *Pesquisa Nacional sobre Acesso, Utilização e Promoção do Uso Racional de Medicamentos* (National Survey on Access, Use and Promotion of Rational Use of Medicines) is an institutional strategy to assess the impact of the implementation of medicines policy in Brazil.

It is necessary to assess whether government efforts to expand the access and guarantee of the benefits of pharmaceutical services to the population are helping to reduce the costs of medicines for families, and whether the resources invested have generated effective impacts on the health of the Brazilian population, such as the appropriate use of medicines in different population strata and major Brazilian regions^i^.

## NATIONAL SURVEY ON ACCESS, USE AND PROMOTION OF RATIONAL USE OF MEDICINES (PNAUM)

The Brazilian Ministry of Health Decree 2077 of September 17, 2013, established the PNAUM, implemented by two coordinated strategies (components): the population component (survey) and the services component (assessment of pharmaceutical services in basic care). The overall objective of the survey is to evaluate the access, use, and rational use of medicines by the Brazilian population and their implementation in SUS basic care.

The survey component of PNAUM, coordinated by Universidade Federal do Rio Grande do Sul, consisted of a cross-sectional nationwide household survey; the first results of this component are presented in this supplement. The component assessing pharmaceutical services in primary care, coordinated by Universidade Federal de Minas Gerais, consisted of a qualitative and quantitative research aimed at evaluating public medicines policy in Brazilian primary health care (the findings shall be presented in a second issue on the theme).

The PNAUM development and consensus process, involving 11 state and federal public universities in different regions of the country, began with the formation of the research team in 2009, and was implemented in September 2013, with initial work in major Brazilian regions and municipalities. Executive oversight was carried out by representatives appointed by the Secretariat of Science, Technology and Strategic Inputs, members of the PNAUM steering committee, throughout the entire survey period, by on-site monitoring, participation in technical meetings and progress reports.

## PERSPECTIVES OF THE BRAZILIAN HEALTH MINISTRY REGARDING PNAUM RESULTS

Based on consolidated and analyzed PNAUM data, the Brazilian Ministry of Health intends to advance and improve the implementation, monitoring and continuous evaluation of Brazilian pharmaceutical policy (National Medicines Policy and PNAF). The findings will make it possible to increase knowledge in the field, establish national indicators on access to and rational use of medicines in Brazil, prioritize strategic paths for policies and programs, assess government efforts in access to and guarantee of pharmaceutical services benefits, and publicize nationally and internationally the results obtained with different actors of society.

## FINAL CONSIDERATIONS

The process of incorporating health technologies requires implementing structured records that enable analysis of impacts generated by such technologies, in line with the growing demands of different Brazilian economic and social sectors^6^.

As stated, the lack of consistent and standardized information and indicators on the evolution of the economic and industrial health care complex, pharmaceutical services, and the field of science, technology and innovation in health is restrictive not only in terms of production and evaluation, but also for the assimilation and consolidation of recommended or agreed indicator standards to support the implementation of policies^8^. Among the negative effects are limitations that prevent the incorporation of the impacts of scientific and technological development on the health of populations on a broader scale^7^.

In the context of this paper, heretofore Brazil had no data produced by nationwide scientific research, shared by academic institutions and health management agencies, exclusively related to pharmaceutical services. This type of evaluation provides potential support to guide government initiatives and public pharmaceutical policies to favor more equitable health action in the country and improve the health conditions and quality of life of the Brazilian population.

This supplement presents the first PNAUM findings on the population component. The survey team has designed the articles based on the specific set of objectives of the survey, in order to answer them. To this end, besides the methodological aspect of PNAUM, the topics addressed will include: global and free access to medicines; use of medicines in older adults, children and people with high blood pressure; use of generic medicines; catastrophic expenditure; self-medication; among others relevant to the area.

The scenario outlined in this article also sought to reflect on the seemingly more structuring measure recently introduced in science, technology and innovation in health under the Brazilian Ministry of Health: an integrating action of the various dimensions of health development, addressing research support, incorporation of technological advances based on scientific grounds, and the level of production development in the economic and industrial health care complex, in order to provide access to health on an universal, integral and equitable basis.

The article also emphasizes a development approach in which, in addition to technical requirements, the concept of innovation also addresses the political and social process to reflect the ethical and social dimension inherent in innovation, which is linked to the fulfillment of human needs, as noted by Celso Furtado:

Development (...) can be defined as a process of social change in which the increasing number of human needs, pre-existing or created by actual change, are met by a differentiation in the production system, generated by the introduction of technological innovations (p. 39-40)^8^.

We therefore invite everyone to read this supplement, comprised of a series of articles considered highly relevant to public health in Brazil, hoping that its content may contribute to greater efficiency in the execution of public pharmaceutical policies, resulting in benefits to the health of the Brazilian population.

## Footnotes

a Brasil. Decreto nº 8.065, de 7 de agosto de 2013. Aprova a Estrutura Regimental e o Quadro Demonstrativo dos Cargos em Comissão e das Funções Gratificadas do Ministério da Saúde e remaneja cargos em comissão. Brasília (DF): 2013 [cited 2015 Jan 29]. Available from: http://www.planalto.gov.br/ccivil_03/_Ato2011-2014/2013/Decreto/D8065.htm

b Country Ranks of SCImago Journal & Country Rank. [cited 2015 Nov 24]. Available from: http://www.scimagojr.com/countryrank.php?area=2700&category=0&region=all&year=2014&order=it&min=0&min_type=it

c SJR SCImago Journal & Country Rank. Country search: Brazil. [cited 2015 Nov 24]. Available from: http://www.scimagojr.com/countrysearch.php?country=BR

d Ministério da Saúde. Pesquisa Saúde: Base de Dados Gerenciais do Decit/SCTIE/MS. Brasília (DF); 2015 [cited 2015 Nov 24]. Available from: http://200.214.130.94/bdgdecit/index.php

e Ministério da Saúde, Conselho Nacional de Saúde. Resolução nº 338, de 6 de maio de 2004. Aprova a Política Nacional de Assistência Farmacêutica e estabelece seus princípios gerais e eixos estratégicos. *Diario Oficial Uniao*. 20 maio 2004; Seção I.

f Brasil. Lei nº 8.080, de 19 de setembro de 1990. Dispõe sobre as condições para a promoção, proteção e recuperação da saúde, a organização e o funcionamento dos serviços correspondentes e dá outras providências. *Diario Oficial Uniao.* 20 set 1990; Seção I:18055.

g Ministério da Saúde. Portaria nº 3.916, de 30 de outubro de 1998. Aprova a Política Nacional de Medicamentos, cuja íntegra consta no anexo desta portaria. *Diario Oficial Uniao*. 10 nov 1998; Seção 1:18-22.

h Ministério da Saúde. Portaria nº 1.214, de 13 de junho de 2012. Institui o Programa Nacional de Qualificação da Assistência Farmacêutica no âmbito do Sistema Único de Saúde (QUALIFAR-SUS). *Diario Oficial Uniao*. 14 jun 2012; Seção I:29-30.

i Costa KS. Acesso e uso de medicamentos: inquéritos de saúde como estratégia de avaliação [these]. Campinas: Faculdade de Ciências Médicas da Universidade Estadual de Campinas; 2014 [cited 2015 Oct 30]. Available from: http://www.bibliotecadigital.unicamp.br/document/?code=000928053
